# Desipramine Protects Neuronal Cell Death and Induces Heme Oxygenase-1 Expression in Mes23.5 Dopaminergic Neurons

**DOI:** 10.1371/journal.pone.0050138

**Published:** 2012-11-27

**Authors:** Hsiao-Yun Lin, Wei-Lan Yeh, Bor-Ren Huang, Chingju Lin, Chih-Ho Lai, Ho Lin, Dah-Yuu Lu

**Affiliations:** 1 Department of Life Sciences, National Chung Hsing University, Taichung, Taiwan; 2 Cancer Research Center, Department of Medical Research, Changhua Christian Hospital, Changhua, Taiwan; 3 Department of Neurosurgery, Buddhist Tzu Chi General Hospital, Taichung Branch, Taichung, Taiwan; 4 Department of Physiology, School of Medicine, China Medical University, Taichung, Taiwan; 5 Department of Microbiology, School of Medicine, China Medical University, Taichung, Taiwan; 6 Graduate Institute of Neural and Cognitive Sciences, China Medical University, Taichung, Taiwan; Emory University, United States of America

## Abstract

**Background:**

Desipramine is known principally as a tricyclic antidepressant drug used to promote recovery of depressed patients. It has also been used in a number of other psychiatric and medical conditions. The present study is the first to investigate the neuroprotective effect of desipramine.

**Methodology/Principal Findings:**

Mes23.5 dopaminergic cells were used to examine neuroprotective effect of desipramine. Western blot, reverse transcription-PCR, MTT assay, siRNA transfection and electrophoretic mobility shift assay (EMSA) were carried out to assess the effects of desipramine. Desipramine induces endogenous anti-oxidative enzyme, heme oxygenase-1 (HO-1) protein and mRNA expression in concentration- and time-dependent manners. A different type of antidepressant SSRI (selective serotonin reuptake inhibitor), fluoxetine also shows similar effects of desipramine on HO-1 expression. Moreover, desipramine induces HO-1 expression through activation of ERK and JNK signaling pathways. Desipramine also increases NF-E2-related factor-2 (Nrf2) accumulation in the nucleus and enhances Nrf2-DNA binding activity. Moreover, desipramine-mediated increase of HO-1 expression is reduced by transfection with siRNA against Nrf2. On the other hand, pretreatment of desipramine protects neuronal cells against rotenone- and 6-hydroxydopamine (6-OHDA)-induced neuronal death. Furthermore, inhibition of HO-1 activity by a HO-1 pharmacological inhibitor, ZnPP IX, attenuates the neuroprotective effect of desipramine. Otherwise, activation of HO-1 activity by HO-1 activator and inducer protect 6-OHDA-induced neuronal death.

**Conclusions/Significance:**

These findings suggest that desipramine-increased HO-1 expression is mediated by Nrf2 activation through the ERK and JNK signaling pathways. Our results also suggest that desipramine provides a novel effect of neuroprotection, and neurodegenerative process might play an important role in depression disorder.

## Introduction

Parkinson’s disease (PD) is a neurodegenerative disorder characterized by the progressive degeneration of dopaminergic neurons of the substantia nigra (SN), giving rise to dopamine depletion in the striatum [1]. The resulting loss of dopaminergic neurons leads to debilitating motor dysfunction including rigidity, resting tremor, mask face and bradykinesia. Although PD is well characterized by motor symptoms, clinical depression is the most common neuropsychiatric disorder in PD patients [2]–[4]. More than 40% of PD patients are observed in depression [5], [6]. Importantly, numerous studies have shown that there is an increased incidence of depression before the onset of PD, and motor fluctuations may greatly affect the occurrence [7], [8]. Depression has been classified as a disorder of the brain and CNS, and is manifested by a combination of symptoms that interferes with the ability to work, study, sleep, eat, and enjoy once pleasurable activities [9]–[12].

Desipramine is a tricyclic antidepressant (TCA), one of an antidepressant drug used to promote recovery of depressed patients. Desipramine does not affect mood or arousal but may cause sedation in non-depressed individuals. However, desipramine exerts a positive effect on mood in depressed individuals. TCAs are potent inhibitors of serotonin and norepinephrine reuptake [13], [14]. It has been reported that desipramine significantly increases anti-apoptotic protein Bcl-2 expression, repairs serotonin and noradrenaline production [15], and prevents stress-induced depressive-like behavioral changes [16]. Interestingly, it has been reported that desipramine is able to reduce MPP^+^-induced cell toxicity in SH-SY5Y, however, despite many studies of those links, the detail molecular mechanisms of antidepressants on neuroprotective effect remain unknown.

HO-1 (also referred to heat-shock protein 32) is a rate-limiting enzyme that catalyzes the degradation of heme, produces biliverdin, iron, and carbon monoxide [17], [18]. In normal brain, the level of HO-1 is rather low [19], but can be strongly induced in response to diverse stress-related cellular stimuli [20], [21], oxidative stress and neuroinflammation [22]–[24]. Therefore, induction of HO-1 contributes to cytoprotection and anti-inflammation [25]–[27], and HO-1 may be a therapeutic target in neurodegenerative diseases and brain inflammation [28]–[30]. In this study, we investigated the neuroprotective effect of antidepressant desipraimine on rotenone- and 6-OHDA-induced neuronal cell death. Our results suggest that desipramine protects rat dopaminergic neurons against cell death through heme oxygenase-1 expression.

## Materials and Methods

### Materials

Desipramine was purchased from Fluka (Buchs, Switzerland). Fetal bovine serum (FBS), Dulbecco’s modified Eagle’s medium/F12 (DMEM/F12), and OPTI-MEM were purchased from Invitrogen-Gibco (Carlsbad, CA). Primary antibodies against β-actin, JNK, ERK2, phospho-ERK1/2, Nrf2 and PCNA were purchased from Santa Cruz Biotechnology (Santa Cruz, CA). Antibody against JNK phosphorylated at Thr 183/185 was purchased from Cell Signaling and Neuroscience (Danvers, MA). ON-TARGET smart pool Nrf2 siRNA, and Control nontargeting pool siRNA were purchased from Dharmacon (Lafayette, CO). 6-OHDA, rotenone, SB203580 and PD98059 were obtained from Sigma–Aldrich (St. Louis, MO). SP600125 was obtained from Tocris Bioscience (Ellisville, MO).

### Cell Cultures

Mes23.5 cell line is a dopaminergic cell line hybridized from murine neuroblastoma-glioma N18TG2 cells with rat mesencephalic neurons, which shows several properties similar to those of primary neurons originated in the substantia nigra [31]. The culture protocol of Mes23.5 was followed our previous report [32]. Briefly, cells were cultured in DMEM/F12 containing Sato’s components growth medium supplemented with 5% FBS at 37°C in a humidified incubator in an atmosphere of 5% CO_2_. Confluent cultures were passaged by trypsinization.

### Western Blot Analysis

The rat Mes23.5 cell line was treated with desipramine for indicated time periods, and whole cell extracts were lysed on ice with radioimmunoprecipitation assay buffer. The nuclear extracts were prepared as described previously [33]. Cells were rinsed with PBS and suspended in hypotonic buffer A for 10 min on ice, and vortexed for 10 s. The lysates were separated into cytosolic and nuclear fractions by centrifugation at 12,000 g for 10 min. The supernatants containing cytosolic proteins were collected. Pellet containing nuclear fraction was re-suspended in buffer C for 30 min on ice.

The supernatants containing nuclear proteins were collected by centrifugation at 13,000 g for 20 min and stored at −80°C. Protein samples were separated by SDS-PAGE (sodium dodecyl sulfate-polyacrylamide) and transferred to PVDF membranes which were then blocked with 5% nonfat milk, and then probed overnight with primary antibody at 4°C. After undergoing PBS washes, the membranes were incubated with secondary antibodies. The blots were visualized by enhanced chemiluminescence using Kodak XOMAT LS film (Eastman Kodak, Rochester, NY). The blots were subsequently stripped through incubation in stripping buffer [34] and reprobed for β-actin as a loading control. Quantitative data were obtained using a computing densitometer and a shareware Image J.

### Reverse Transcription-PCR (RT-PCR)

Total RNA was extracted from Mes23.5 cell line using a TRIzol kit (MDBio Inc., Taipei, Taiwan). The reverse transcription reaction was performed using 2 µg of total RNA that was reverse transcribed into cDNA with the oligo(dT) primer. After preincubation at 50°C for 2 min and 95°C for 10 min, the PCR was performed as 30 cycles of 95°C for 10 s and 60°C for 1 min. The threshold was set above the non-template control background and within the linear phase of target gene amplification to calculate the cycle number at which the transcript was detected (denoted as CT). The oligonucleotide primers were

HO-1:

5′- TCTAT CGTGC TCGCA TGAAC-3′ and

5′-CAGCT CCTCA AACAG CTCAA -3′

GAPDH:

5′-CTCAA CTACA TGGTC TACAT GTTCC A-3′ and

5′-CTTCC CATTC TCAGC CTTGA CT-3′

### Measurement of Cell Viability

Cell viability was assessed by the 3-(4,5-dimethylthiazol-2-yl)-2,5-diphenyltetrazolium bromide (MTT) assay. Cells cultured in 96-well plates were pre-treated with various inhibitors before treated with desipramine for 8 h followed by treatment with rotenone or 6-OHDA. After treatment, MTT (0.5 µg/ml) was added for 60 min, the culture medium was then removed, cells were dissolved in dimethyl sulfoxide and shaken for 10 min. OD values at 550 nm were measured by microplate reader. The absorbance indicates the enzymatic activity of mitochondria and provides information of cell viability.

### Sulphorhodamine B Assay

Cell viability was assessed by the sulphorhodamine B (SRB) assay. Cells cultured in 96-well plates were treated with various inhibitors before treated with desipramine for 8 h followed by stimulation with rotenone or 6-OHDA. After treatment, the culture medium was then removed and cells were fixed in situ by gentle addition of 50 µl per well of 10% TCA and plates were incubated for 1 h at 4°C. After discarding supernatant, plates were then washed for 5 times with PBS, and then added 50 µl of SRB solution to each well, and plates were incubated for 15 min at room temperature. After staining, cells were washed 5 times with 1% acetic acid and dissolved in tris-base. After 10 mins’ shaking, OD values at 515 nm were measured by microplate reader. The absorbance provides information of cell viability.

### Trypan Blue Exclusion Assay

The trypan blue exclusion assay was used to assess cell viability after 6-OHDA or rotenone exposure. Culture medium was removed and 0.2% trypan blue in TBS was added to the cells for 10 min. After the removal of the trypan blue solution, cells were fixed with 4% paraformaldehyde in PBS for 5 min, then replaced by TBS. Cells were then observed under bright field microscope.

### Immunocytofluorescent Staining

Cells were seeded onto glass coverslips and exposed to desipramine for 2 h, then washed with PBS and fixed with 4% paraformaldehyde for 15 min, after which they were permeabilized with Triton X-100 for 30 min. After blocking with 5% nonfat milk in PBS buffer, cells were incubated with rabbit anti-Nrf2 antibody for 1 h at room temperature. After a brief wash, cells were incubated with a secondary antibody conjugated with Alexa 488-Flour (1∶200; Invitrogen Life Technologies, Carlsbad, CA). Finally, cells were washed again, mounted, and visualized with fluorescence microscope. Quantitative data were obtained using a computing densitometer and Image J.

### Transfection

Mes23.5 cells were grown to 80% confluent in 6-well plates and transfected with control siRNA or Nrf2 siRNA on the following day by Lipofectamine™ 2000 (LF2000; Invitrogen). Control siRNA or Nrf2 siRNA and LF2000 were premixed in OPTI-medium for 20 min and applied to the cells (0.8 ml/well). Medium containing 5% FBS (0.8 ml) was added 4∼6 h later. After 24 hrs’ transfection, LF2000-containing medium was replaced with DMEM/F12 medium containing 2% FBS and treated with desipramine for another 24 h.

### Electrophoretic Mobility Shift Assay (EMSA)

The electrophoretic mobility shift assay gel shift kit (Panomics, Redwood City, CA) was used according to our previous report [35]. Nuclear extract (4 µg) of Mes23.5 was incubated with poly d(I-C) at room temperature for 5 min. The nuclear extract was then incubated with biotin-labeled probes at room temperature for 30 min. After electrophoresis on an 8% polyacrylamide gel, the samples on gel were transferred onto a presoaked Immobilon-Nyt membrane (Millipore, Billerica, MA). The membrane was cross-linked in an oven for 1 min and then developed with blocking buffer and streptavidin–horseradish peroxidase conjugate before being subjected to Western blot analysis.

### Statistics

Statistical analysis was performed using software Graphpad Prism 4.01 (Graph Pad Software Inc., San Diego, CA). The values given are means ± S.E.M. Statistical analysis between two samples was performed using Student’s *t-*test. Statistical comparisons of more than two groups were performed using one-way ANOVA with Bonferroni’s post hoc test. In all cases, a value of p less than 0.05 was considered significant.

## Results

### Desipramine and Fluoxetine Induce HO-1 Expression in Mes23.5 Cells

**Figure 1:**
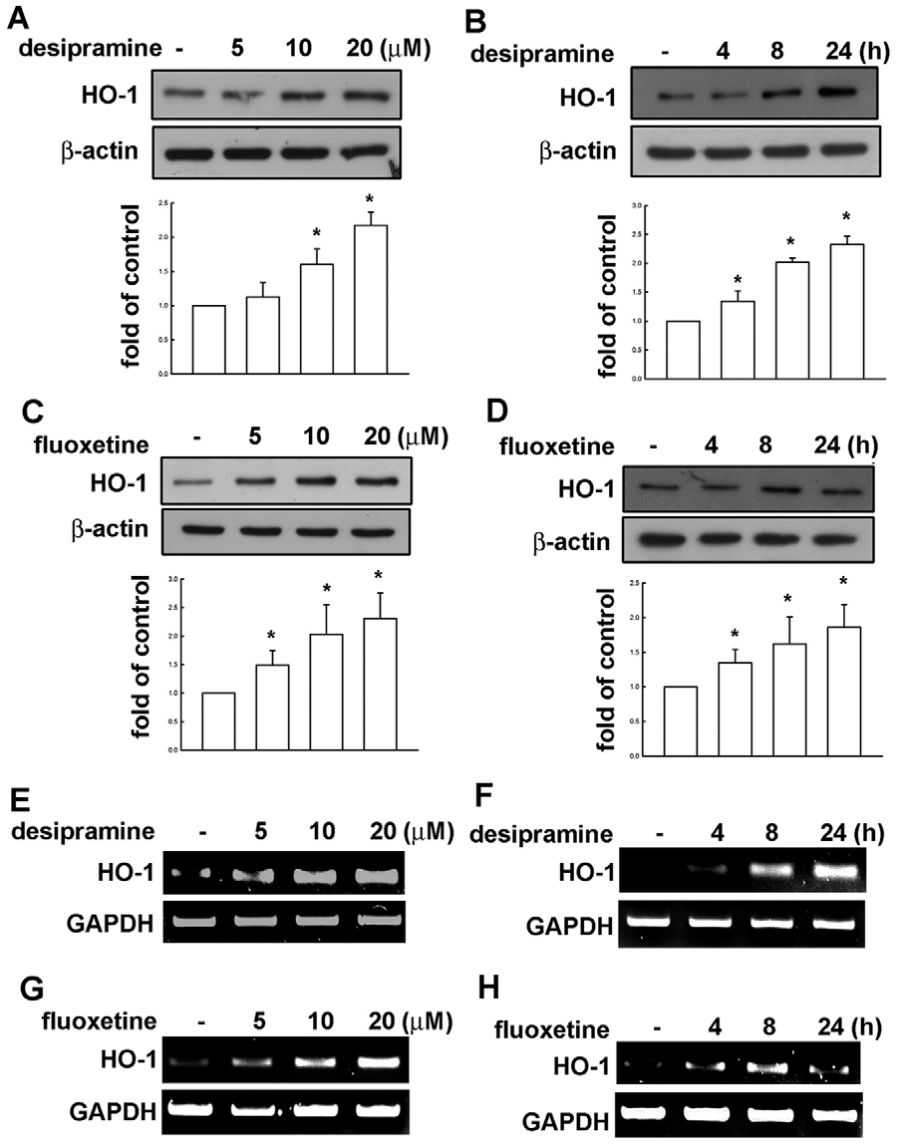
Desipramine and fluoxetine increase HO-1 expression in Mes23.5 dopaminergic neurons. Cells were incubated with various concentrations (5, 10 or 20 µM) of desipramine (A) or fluoxetine (C) for 24 h or with desipramine (20 µM; B) or fluoxetine (20 µM; D) for the indicated time periods (4, 8 and 24 h). Whole cell lysates were extracted and subjected to Western blot for detection of HO-1 expression. The HO-1 expression is significantly different between control group and desipramine or fluoxetine treatment groups (one-way ANOVA followed by Bonferroni’s post hoc test). Results are expressed as the means ± S.E.M. from three independent experiments. *, *p*<0.05 as compared with the vehicle control group. Cells were incubated with various concentrations (5, 10 or 20 µM) of desipramine (E) or fluoxetine (G) for 8 h or with desipramine (20 µM; F) or fluoxetine (20 µM; H) for indicated time periods (4, 8 and 24 h). The quantitative data are shown in below. HO-1 mRNA expression was determined by RT-PCR. Results are the representatives of three independent experiments.

We recently reported that Omega-3 polyunsaturated fatty acids [32] and berberine [15] increase HO-1 expression and exert anti-neuroinflammatory responses in glial cells. Firstly, we identified the effect of desipramine on the regulation of HO-1 in Mes23.5 dopaminergic neurons. Stimulation of desipramine with various concentrations (5, 10, or 20 µM for 24 h) and for indicated time periods (0, 4, 8 or 24 h) increased HO-1 protein expression (Fig. 1A and 1B). Moreover, desipramine also increased HO-1 mRNA expression in time-dependent and concentration-dependent manners (Fig. 1E and 1F). We further analyzed whether a different type of antidepressant SSRI fluoxetine regulates HO-1 expression. Stimulation of fluoxetine with various concentrations (5, 10, or 20 µM for 24 h) and for indicated time periods (0, 4, 8 or 24 h) also increased HO-1 protein expression (Fig. 1C and 1D). In addition, fluoxetine also increased HO-1 mRNA up-regulation (Fig. 1G and 1H).

### ERK, JNK Signaling Pathways are Involved in Desipramine-mediated HO-1 up-regulation

**Figure 2:**
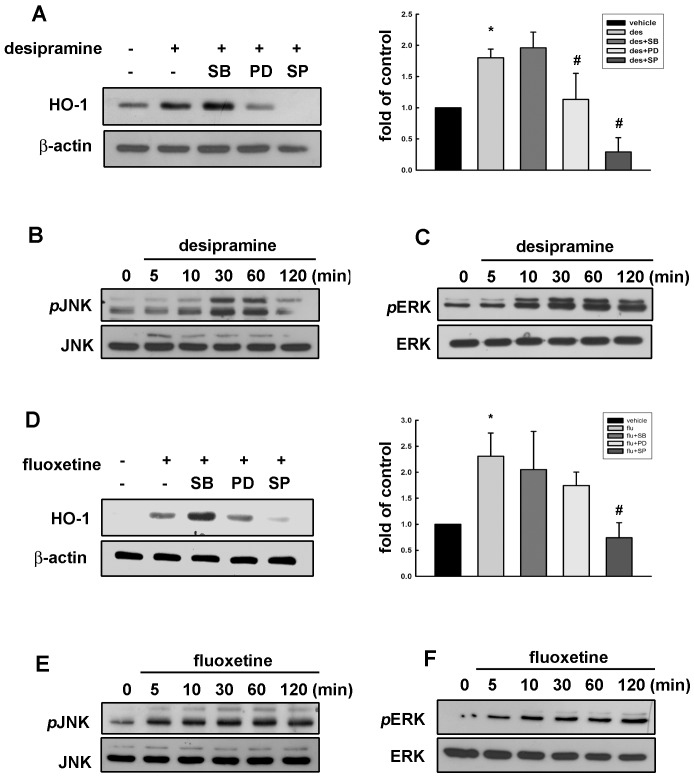
ERK and JNK signaling pathways are involved in desipramine- and fluoxetine-increased HO-1 expression in Mes23.5 dopaminergic neurons. Cells were pretreated with various of MAP kinase inhibitors SB203580 (10 µM), PD98059 (20 µM) or SP600125 (10 µM) for 30 min followed by stimulation with desipramine (20 µM; A) or fluoxetine (20 µM; D) for another 24 h. Whole cell lysates were subjected to Western blot for detection of HO-1 expression. Cells were incubated with desipramine or fluoxetine for the indicated time periods, and cell lysates were subjected to immunoblots with antibodies against phospho-JNK (B and C) and phospho-ERK (D and E). Results are the representatives of three or four independent experiments.

MAP kinase pathways are the most important signaling pathways that participate in transducing a variety of biological responses. Numerous evidences reported that activation of MAP kinase pathways contribute to regulation of HO-1 expression [36]–[38]. Therefore, we examined the effect of desipramine on the activation of MAP kinases in Mes23.5 dopaminergic neurons. As shown in Figure 2A, desipramine-increased HO-1 expression could be inhibited by ERK (PD98059) and JNK (SP600125) inhibitors, but not inhibitor of p38 (SB203580). Desipramine also resulted in the JNK and ERK phosphorylation initiated at 10 min and sustained to 120 min (Fig. 2B and 2C). Similarly, treatment of JNK inhibitor SP600125 also effectively reduced fluoxetine-induced HO-1 expression (Fig. 2D). Stimulation of cells with fluoxetine also increased JNK and ERK phosphorylation time-dependently (Fig. 2E and 2F).

### Involvement of Nrf2 Activation in Desipramine-induced HO-1 Expression in Mes23.5 Dopaminergic Neurons

**Figure 3:**
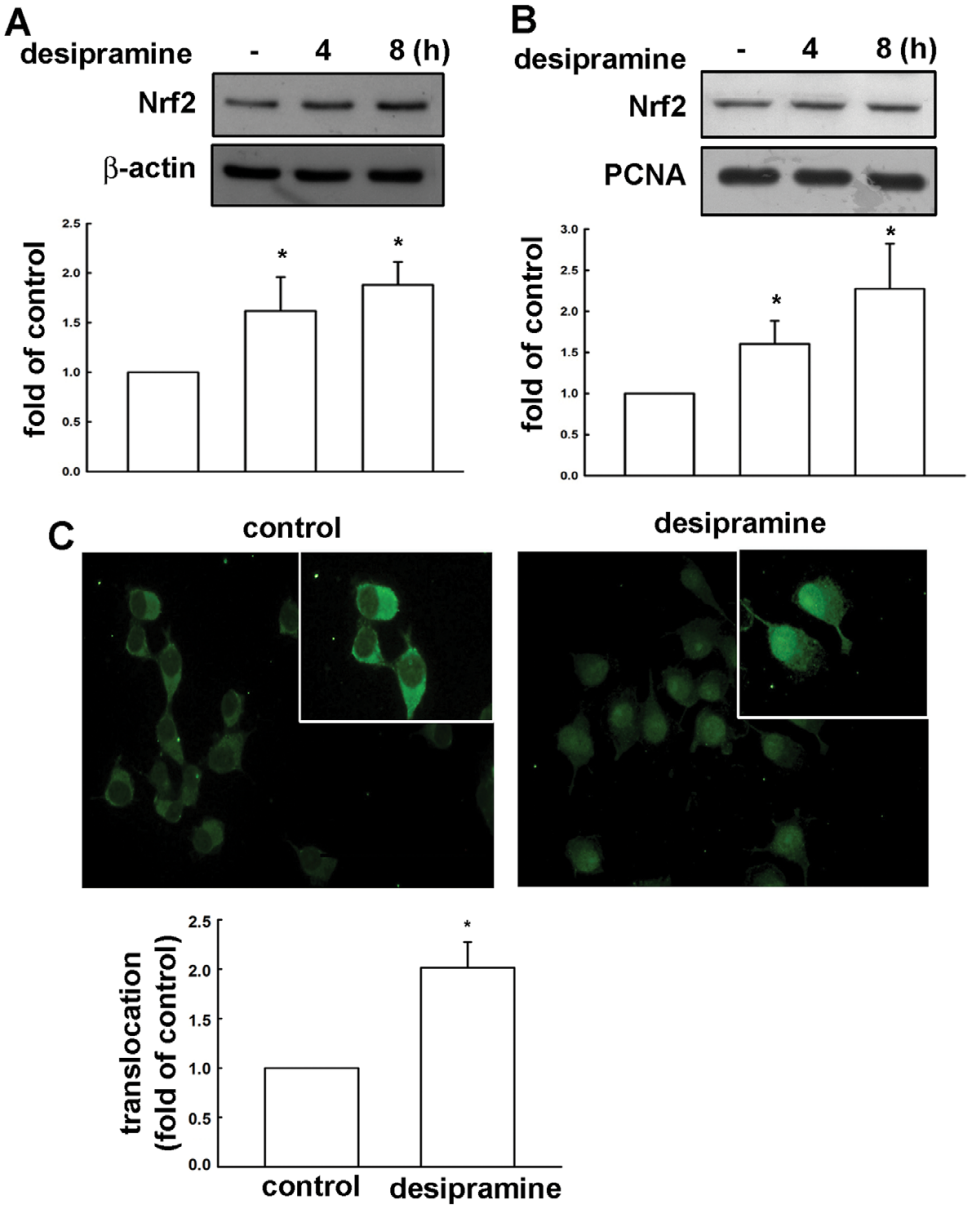
Desipramine induces Nrf2 translocation from cytoplasm to nucleus in Mes23.5 dopaminergic neurons. Cells were incubated with desipramine (20 µM) for indicated time periods (4 or 8 h), and Nrf2 expression levels in whole cell lysates (A) and nuclear extracts (B) were determined by immunoblotting with Nrf2-specific antibody. PCNA was used as the loading control for nuclear fraction. The quantitative data are shown in below. The Nrf2 expression is significantly different between desipramine treatment group and control group in nuclear extract (one-way ANOVA followed by Bonferroni’s post hoc test). Results are expressed as the means ± S.E.M. from four independent experiments. *, *p*<0.05 as compared with the vehicle control group. Cells were treated with or without desipramine (20 µM) for 2 h, and the levels of Nrf2 were determined by immunoflourescence (C). Note that the Nrf2 translocates from cytoplasm to nucleus in response to desipramine stimulation. Results are the representatives of three independent experiments.

**Figure 4:**
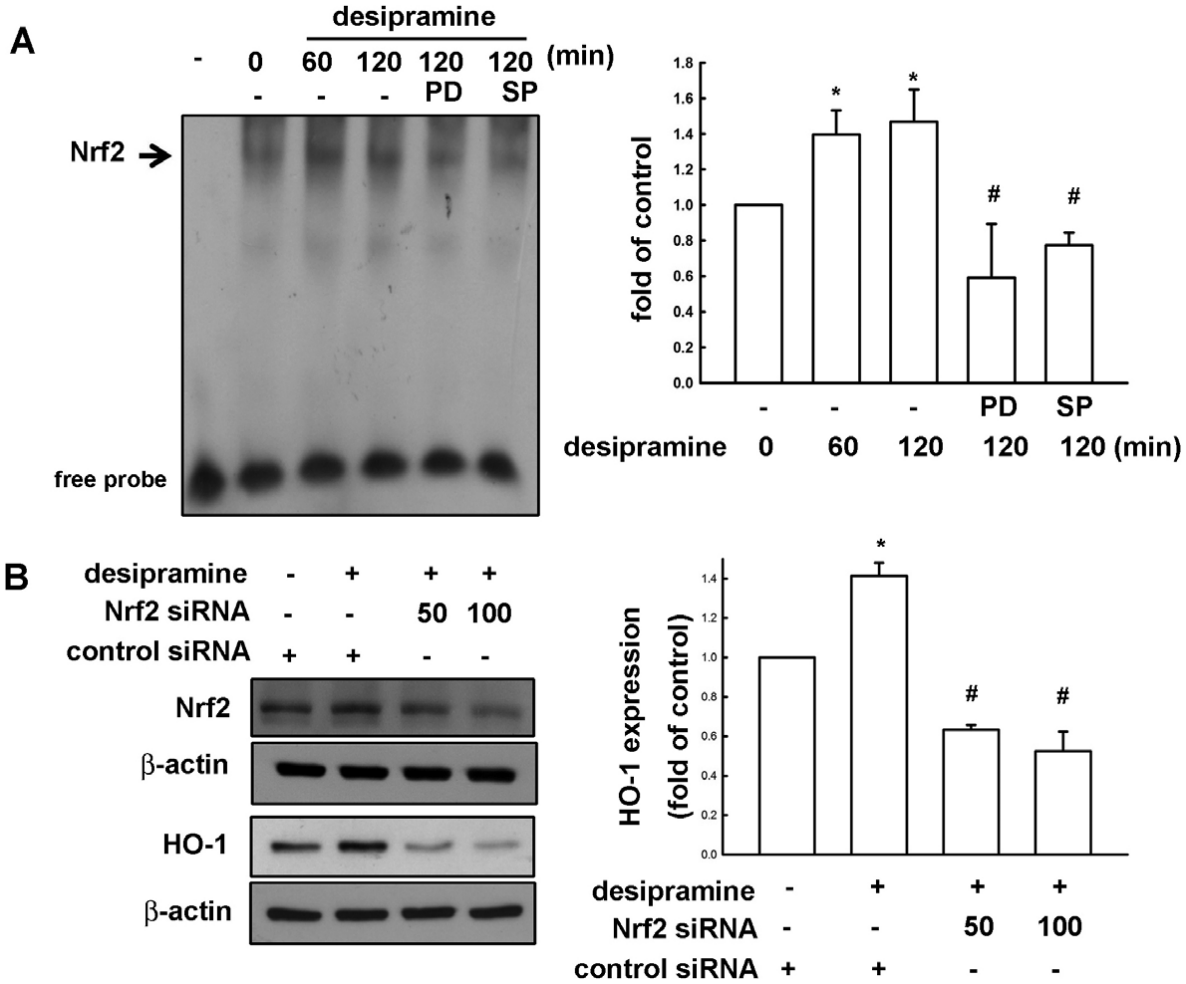
Desipramine increases HO-1 expression through Nrf2 activation in Mes23.5 dopaminergic neurons. (A) Cells were treated with desipramine (20 µM) for indicated time periods (60 or 120 min) and nuclear extracts were collected, and the binding activity of Nrf2 to Nrf2-DNA binding element was examined by EMSA analysis. The DNA binding activity of Nrf2 is significantly different between desipramine treatment group and control group (one-way ANOVA followed by Bonferroni’s post hoc test). Cells were pretreated with PD98059 or SP600125 with desipramine (20 µM), and nuclear extracts were examined by EMSA analysis. Lane 1 was loaded without nuclear extracts (probe only). Results are expressed as the means ± S.E.M. from three independent experiments. *, *p*<0.05 as compared with the vehicle control group. #, p<0.05 as compared with the desipramine treatment group. (B) Cells were transfected with Control siRNA (100 nM) or Nrf2 siRNA (50 and 100 nM) for 24 h followed by stimulation with desipramine (20 µM) for another 24 h, and the protein levels of Nrf2 and HO-1 were determined by Western blot. The HO-1 expression is significantly different between Nrf2 siRNA group and control siRNA group (one-way ANOVA followed by Bonferroni’s post hoc test). Results are expressed as the means ± S.E.M. from three independent experiments. *, *p*<0.05 as compared with the control group. #, p<0.05 as compared with the desipramine treatment alone group.

Numerous evidences reported that stress response element (StRE)/Nrf2 transcription factor pathway is an important transcriptional factor for HO-1 expression [39]–[41]. We therefore examined whether the Nrf2 signaling is involved in desipramine-induced HO-1 expression. Treatment of Mes23.5 dopaminergic neurons with desipramine enhanced Nrf2 expression time-dependently (Fig. 3A), and desipramine also resulted in an accumulation of Nrf2 in nucleus (Fig. 3B). Immunofluorescence staining of Nrf2 localization also showed that Nrf2 translocated from cytoplasm to nucleus in response to desipramine treatment for 2 h (Fig. 3C). Moreover, to evaluate desipramine-mediated Nrf2 transcription factor binding activity in Mes23.5 cells, EMSA were performed to test DNA binding activity. Stimulation of cells with desipramine for 1 or 2 h increased the DNA binding activity of Nrf2 in nuclear extracts, treatment with PD98059 or SP600125 reduced desipramine-increased DNA binding activity of Nrf2 (Fig. 4A). There was no detectable DNA binding complex without loading nuclear protein. To investigate whether the desipramine-induced HO-1 expression is mediated through Nrf2 activation, cells were transfected with Nrf2 siRNA for 24 h followed by stimulation with desipramine. Transfection with Nrf2 siRNA for 24 h in Mes23.5 dopaminergic neurons reduced Nrf2 expression. Moreover, tranfection with Nrf2 siRNA also significantly reduced desipramine-induced HO-1 expression in a concentration-dependent manner (Fig. 4B). These results suggest that desipramine-increased HO-1 expression was mediated through ERK, JNK and Nrf2 pathways in Mes23.5 dopaminergic neurons.

### Desipramine-induced HO-1 Expression Protects Mes23.5 Cells from 6-OHDA or Rotenone-induced Neurotoxicity

**Figure 5:**
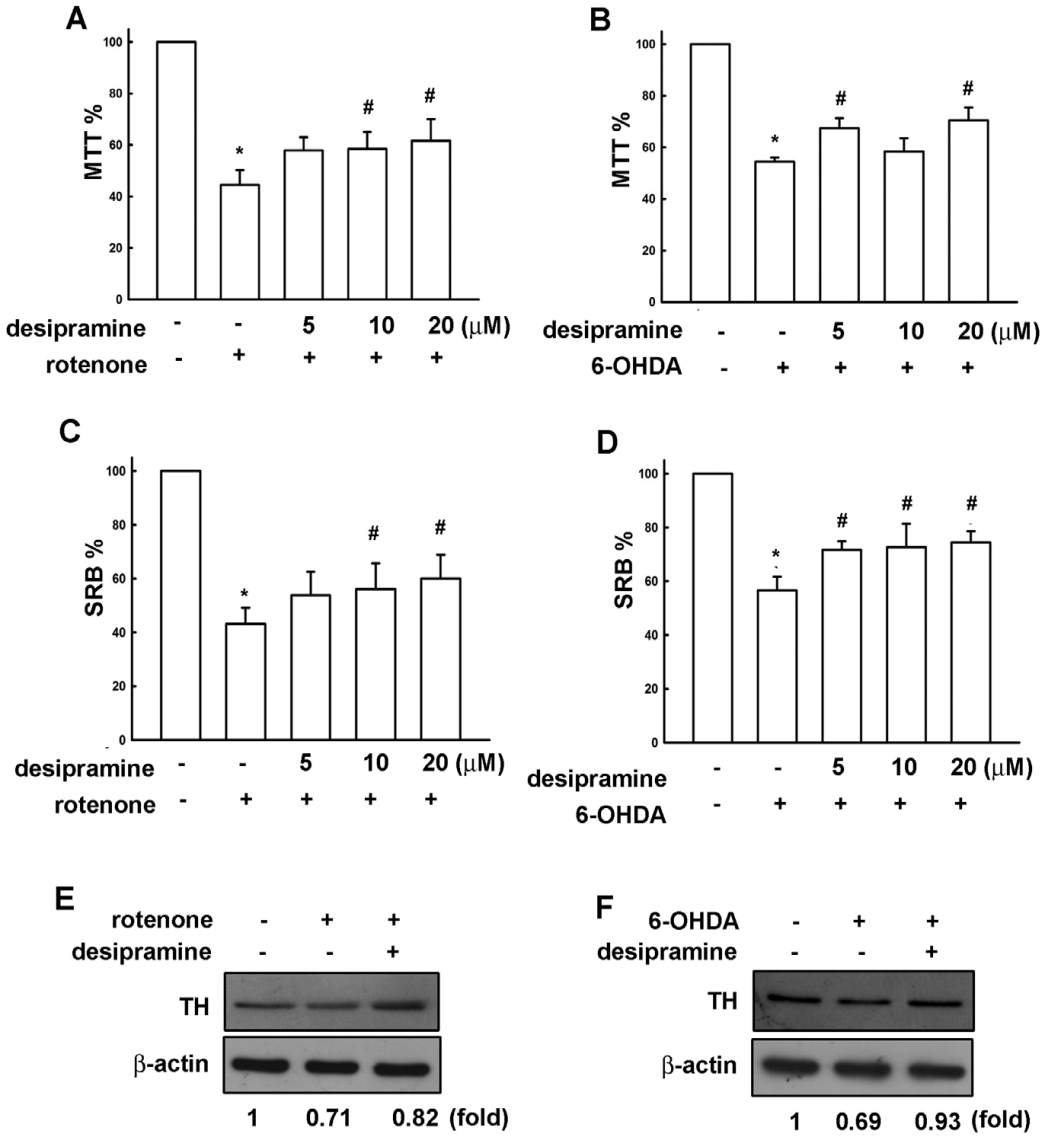
Neuroprotection of desipramine on rotenone- and 6-OHDA-induced neurotoxicity. Mes23.5 dopaminergic neurons were pretreated with various concentrations of desipramine for 8 h and followed by treatment with rotenone (3 µM) for another 16 h. The cell viability was determined by MTT assay and SRB assay (A and C, respectively). The neuroprotective effects are significantly different between rotenone alone group and rotenone treated with desipramine group in both MTT and SRB assays (one-way ANOVA followed by Bonferroni’s post hoc test). Results are expressed as the means ± S.E.M. from four independent experiments. *, *p*<0.05 as compared with the vehicle group. #, *p*<0.05 as compared with the desipramine-treated group. Cells were pretreated with various concentrations of desipramine for 8 h and followed by treatment with 6-OHDA (50 µM) for another 16 h. The cell viability was determined by MTT assay and SRB assay (B and D, respectively). The neuroprotective effect is significantly different between 6-OHDA alone group and 6-OHDA treated with desipramine group in SRB assay (one-way ANOVA followed by Bonferroni’s post hoc test). Results are expressed as the means ± S.E.M. from four independent experiments. *, *p*<0.05 as compared with the vehicle group. #, *p*<0.05 as compared with the desipramine-treated group. Whole cell lysates were subjected to Western blot for detection of tyrosine hydroxylase (TH). Results are the representatives of at least three independent experiments.

**Figure 6:**
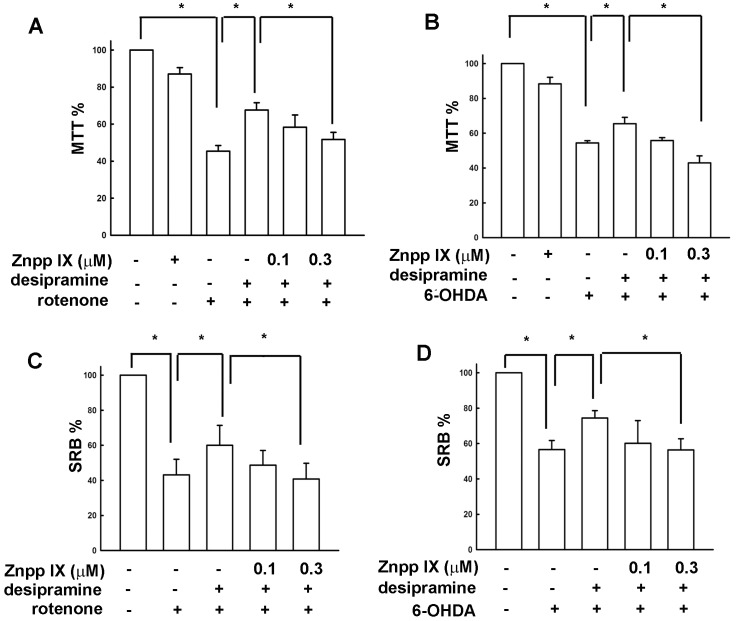
Involvement of HO-1 in desipramine-mediated neuroprotective effect in Mes23.5 dopaminergic neurons. Cells were treated with ZnPP IX (0.1 or 0.3 µM) for 30 min and follow by treatment with desipramine for 8 h, and then treated with rotenone (A and C) or 6-OHDA (B and D) for another 16 h. Results are expressed as the means ± S.E.M. from four independent experiments. *, *p*<0.05 as compared with vehicle group.

**Figure 7:**
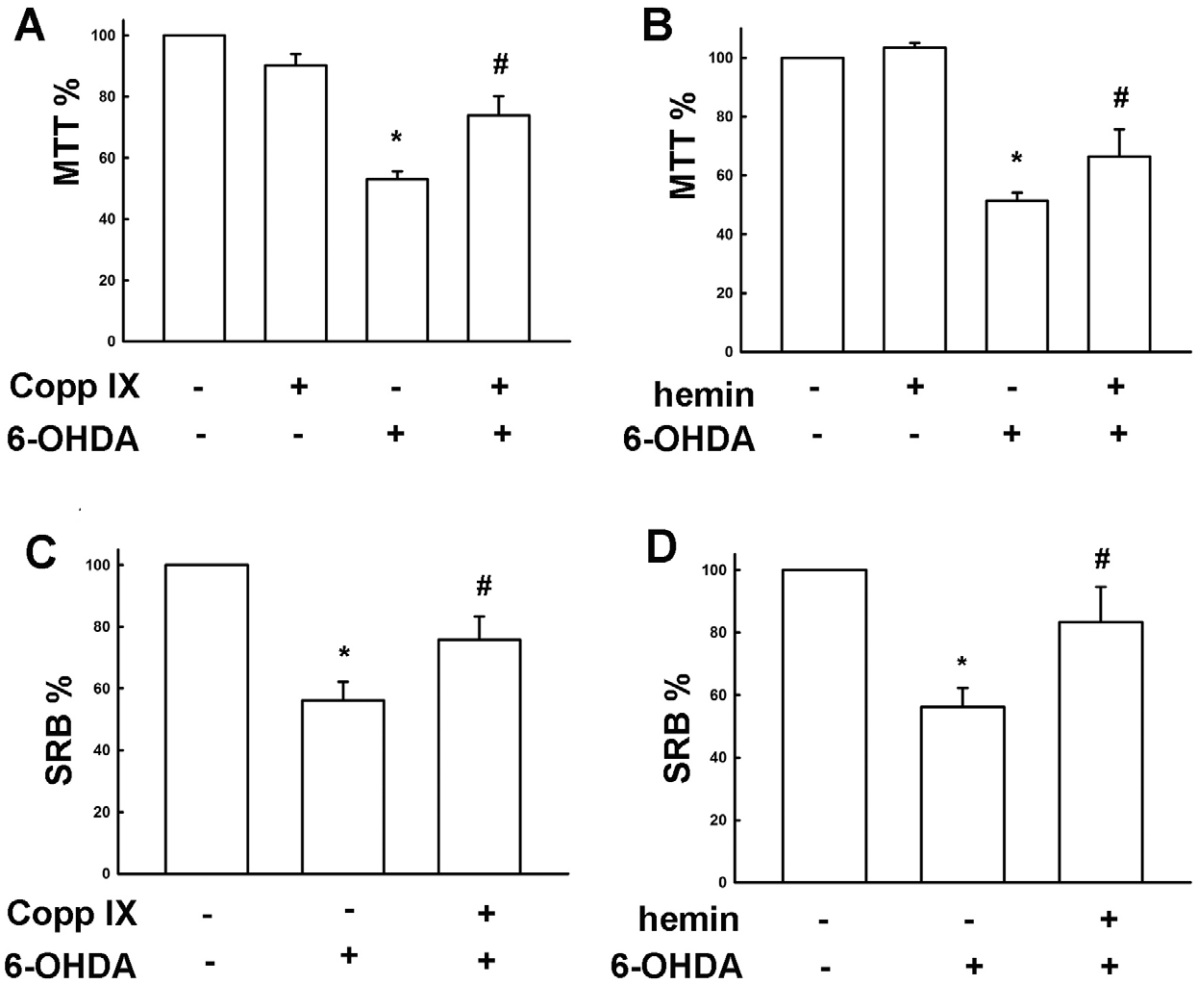
Role of HO-1 in neuroprotective effect in Mes23.5 dopaminergic neurons. Cells were treated with Copp IX (1 µM) (A and C) or hemin (B and D) for 8 h followed by treatment with 6-OHDA (50 µM) for another 16 h. Cell viability was determined by MTT assay and SRB assay. Results are expressed as the means ± S.E.M. from four independent experiments. *, *p*<0.05 as compared with vehicle group. #, *p*<0.05 as compared with desipramine-treated group.

Rotenone and 6-OHDA are useful neurotoxins as resulting in dopaminergic neuron degeneration [42]–[45]. Studies using neurotoxins have provided insights into the molecular mechanisms of dopaminergic neuronal death. To ensure the actual cell loss was correlated with the cell death assay values, a cell count assay had been evaluated. The Mes23.5 dopaminergic neurons’ cell death induced by rotenone and 6-OHDA were correlated with MTT assay value in a concentration-dependent manner (Figure S1). Surprisingly, pretreatment of desipramine at 20 µM followed by rotenone (Fig. 5A and 5C) and 6-OHDA (Fig. 5B and 5D) dramatically protected cells from neurotoxicity. The neuroprotective effect of desipramine exerts only by pretreatment, but not co-treatment or posttreatment with the neurotoxins (Figure S2). Moreover, pretreatment with desipramine also reversed rotenone- and 6-OHDA-induced tyrosine hydroxylase (TH) loss in Mes23.5 dopaminergic neurons (Fig. 5E and 5F). To further confirm the role of desipramine-induced HO-1 expression on neuroprotective effect, we examined whether desipramine protects neuronal cell death through HO-1 up-regulation. Inhibition of HO-1 activity by a HO-1 pharmacological inhibitor Znpp IX attenuated desipramine-protected neuronal cell death determined by MTT (Fig. 6A and 6B) and SRB (Fig. 6C and 6D) assay. Otherwise, HO-1 activators cobalt protoporphyrin IX (CoPP IX) (Fig. 7A and 7C) and hemin (Fig. 7B and 7D) also protected Mes23.5 cells from 6-OHDA-induced neurotoxicity.

## Discussion

Clinical studies have suggested that depression and PD are closely related [46]–[48], and depression is a negative impact on the quality of life of PD patients and their families. Dopamine replacement therapy, especially with higher doses of levodopa, is sometimes accompanied by depression [49]. Moreover, the presence of levodopa-induced dyskinesia is correlated with an increased incidence of depression [50]. Furthermore, a number of PD patients who had subthalamic nucleus deep brain stimulation suffered from behavioral side effects including cognitive impairments and depression [51]–[53]. Treatment with TCAs is restricted by adverse effects, however, TCAs like desipramine are effective in treating depression in PD patients and may even reduce motor symptoms [54]. In this study, we used neurotoxins rotenone and 6-OHDA to induce dopaminergic neuron toxicity that mimics human neurodegenerative disease and neuropathological representative of PD. Previous report has reported the antioxidant effects of antidepressant agents [55]. Furthermore, it has also been reported that desipramine and fluoxetine protect neurons against microglial activation-mediated neurotoxicity [56]. However, the mechanism of antidepressants on neuroprotective effect remains unknown.

Desipramine is one of the most common tricyclic antidepressant (TCA) used to promote recovery of depressed patients. For adults, the therapeutic concentration of desipramine is approximately 1 µM [57]. Recently, it has been reported that a higher concentration of desipramine (up to 390 µM) exerts neuroprotective effect in primary culture [58]. Importantly, fluoxetine accumulates in the human brain relative to plasma, with brain concentrations ranging up to 30 µM [59]. Here, we showed that desipramine and fluoxetine at concentrations up to 20 µM increase HO-1 expression and protect neuronal cell death. Hence, explore the therapeutic potential of antidepressants may help us to identify target molecules for drug development and therapy against neurodegeneration. To ascertain the significance of their neuroprotective effects in humans, further clinical trials with antidepressants are required as well as retrospective epidemiological studies assessing the prevalence of neurodegenerative diseases. The observation that antidepressants have neuroprotective properties might have important clinical implications since these drugs are heavily prescribed worldwide and chronic treatment often lasts several months.

It has been reported that HO-1 plays a significant role in anti-apoptosis, anti-oxidant and neuroprotection [50], [60]–[63]. Numerous evidences have showed that neuronal and non-neuronal cells increase the synthesis of HO-1 under oxidative injury and inflammation conditions, which plays an important role in neuroprotective function [64], [65]. We have also reported that increased HO-1 expression in glial cells may contribute to anti-neuroinflammatory responses and exert neuroprotection [15], [32]. Importantly, the expression of anti-oxidative enzyme HO-1 exerts neuroprotective effect by protecting dopaminergic neurons [66]–[69] and might characterize the antidepressant mechanisms [32], [49], [70]–[72]. Previous study also reveal that fluoxetine, an antidepressant of the SSRIs class, attenuates brain injury via enhancement of HO-1 expression [73]. ERK and JNK pathways are also associated with various plasticity processes including neurogenesis, axonal growth, and regulation of BDNF levels [74]. Alteration of the MAPK pathways such as ERK expression has been studied in post-mortem samples of depression patients and animal models [75]–[77]. Furthermore, previous report also shown that there is different potency of ERK activation between desipramine and fluoxetine [78]. In present study, the enhancement of HO-1 expression by desipramine could be regulated by ERK and JNK, but the effect of fluoxetine was only regulated by JNK. Moreover, it has also been reported that the difference in potency between desipramine and fluoxetine exerts in many functions, such as inhibition of chemotaxis on polymorphonuclear leukocytes [79], acute hypnotic/sedative effect of ethanol [80] and development of ethanol-induced behavioral sensitization [81]. Our results reveal that desipramine-enhanced HO-1 expression sustains to 24 h, however, fluoxetine-increased HO-1 expression reaches a peak at 8 h. Our results and previous reports indicate that different types of antidepressant exert different molecular mechanisms which could provide a novel view for clinical drug choice.

It has been reported that Nrf2 may be a new drug target of treating depression [82]. Nrf2 is known as an important transcription factor involves in antioxidant response, binding to antioxidant response elements (ARE) encoding detoxification enzymes such as HO-1 [83]. Moreover, it has also been reported that activation of Nrf2 provides the insight of treatment of depression and neurodegenerative disease [84], [85]. Previous reports have shown that ERK and JNK pathways are involved in the Nrf2 activation and HO-1 up-regulation in various cell types [86], [87]. Here, our study also showed that desipramine increases HO-1 expression through ERK and JNK pathways, leading to Nrf2 activation. In line with our results, we also found that fluoxetine-induced HO-1 up-regulation is mediated by ERK and JNK pathways in Mes23.5 dopaminergic neurons. Our results demonstrated that pretreatment of desipramine protects neuronal cell death in dopaminergic neurons through HO-1 up-regulation. The neuroprotective effect of desipramine exerts only by pretreatment, but not co-treatment or posttreatment with the neurotoxin. It suggests that desipramine-increased HO-1 expression and protecting neuronal cell death might require a de novo synthesis pathway. Moreover, our study also showed that HO-1 inducer and activator effectively protect dopaminergic neurons from cell death. These findings indicate that when neuronal cells have adequate or enhanced levels of anti-oxidative agents, they can protect themselves from oxidative damage. Neuroprotection of dopaminergic neurons by the activation of Nrf2 in PD is also regarded as a promising therapeutic approach.

In conclusion, our study elucidates the neuroprotection effects of desipramine and the regulatory molecular mechanisms of desipramine-induced HO-1 expression through the ERK and JNK signaling pathways in Mes23.5 dopaminergic neurons. This is the first study reporting antidepressant against neuronal cell death. Connecting the evidence pointing to depression in PD and clinical studies will support exploring the novel therapeutic potential of desipramine and fluoxetine in treating neurodegeneration.

## Supporting Information

Figure S1 **Effect of rotenone- and 6-OHDA-induced neurotoxicity in Mes23.5 dopaminergic neurons.** Cells were treated with various concentrations of 6-OHDA (25, 50, 75, or 100 µM; A) or rotenone (1, 3, 5, or 10 µM; B) for 24 h. The cell viability was determined by MTT assay and cell number count. Results are expressed as the means ± S.E.M. from four independent experiments. Note that the cell viability do not have significant difference between MTT assay and cell number count in 6-OHDA treatment (up to 50 µM) and rotenone treatment (up to 10 µM).
(TIF)

Figure S2 **Protective effect of desipramine on rotenone- and 6-OHDA-induced neurotoxicity.** Mes23.5 cells were treated with desipramine before (0 or 5 h) or after (5 h) rotenone (3 µM; A) or 6-OHDA (50 µM; B) for another 16 h. The cell viability was determined by MTT assay. Results are expressed as the means ± S.E.M. from four independent experiments. *, *p*<0.05 as compared with the vehicle control group. #, *p*<0.05 as compared with the rotenone- or 6-OHDA-treated group.
(TIF)
