# Supplementary figures and images for: Desipramine Protects Neuronal Cell Death and Induces Heme Oxygenase-1 Expression in Mes23.5 Dopaminergic Neurons
Source: PLoS One. 2012 Nov 27;7(11):e50138. doi: 10.1371/journal.pone.0050138 (PMC3507930; doi:10.1371/journal.pone.0050138)

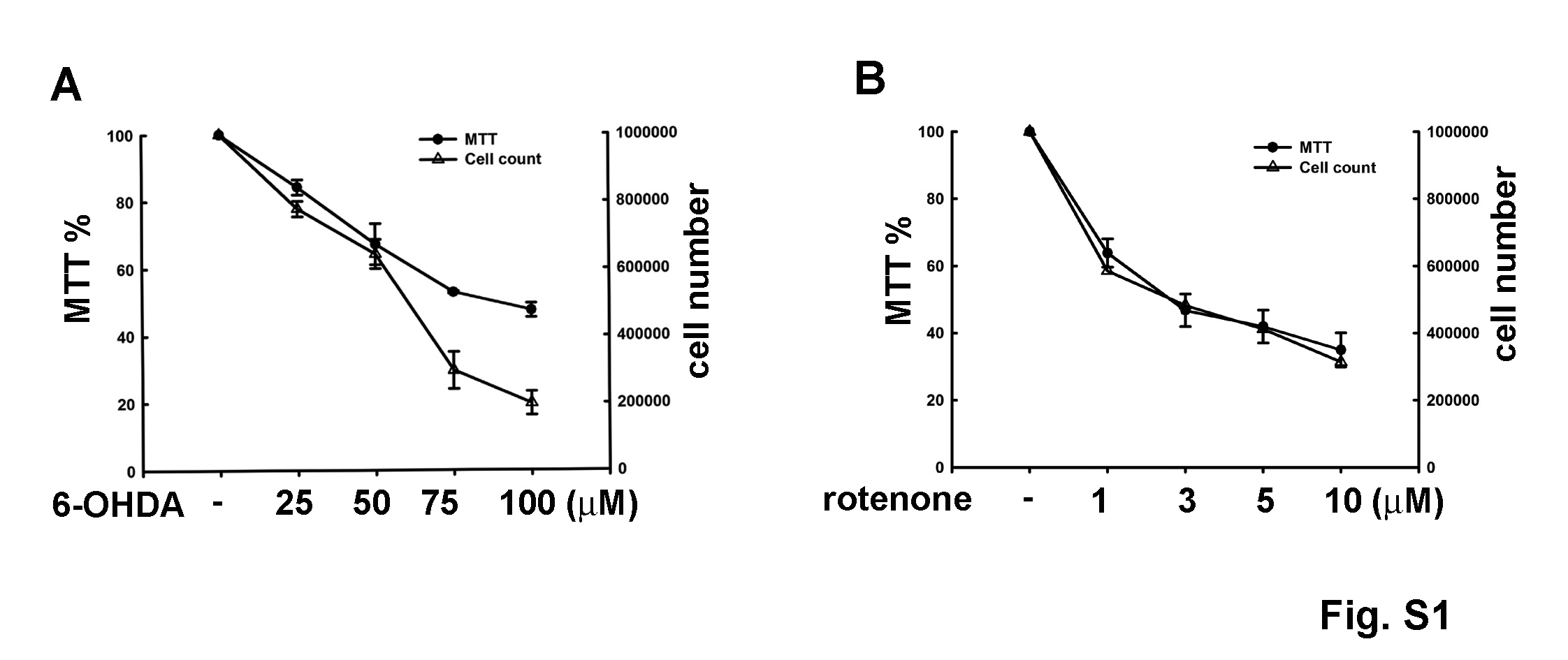

Supplement: Figure S1 — Effect of rotenone- and 6-OHDA-induced neurotoxicity in Mes23.5 dopaminergic neurons. Cells were treated with various concentrations of 6-OHDA (25, 50, 75, or 100 µM; A) or rotenone (1, 3, 5, or 10 µM; B) for 24 h. The cell viability was determined by MTT assay and cell number count. Results are expressed as the means ± S.E.M. from four independent experiments. Note that the cell viability do not have significant difference between MTT assay and cell number count in 6-OHDA treatment (up to 50 µM) and rotenone treatment (up to 10 µM). (TIF) [file pone.0050138.s001.tif]

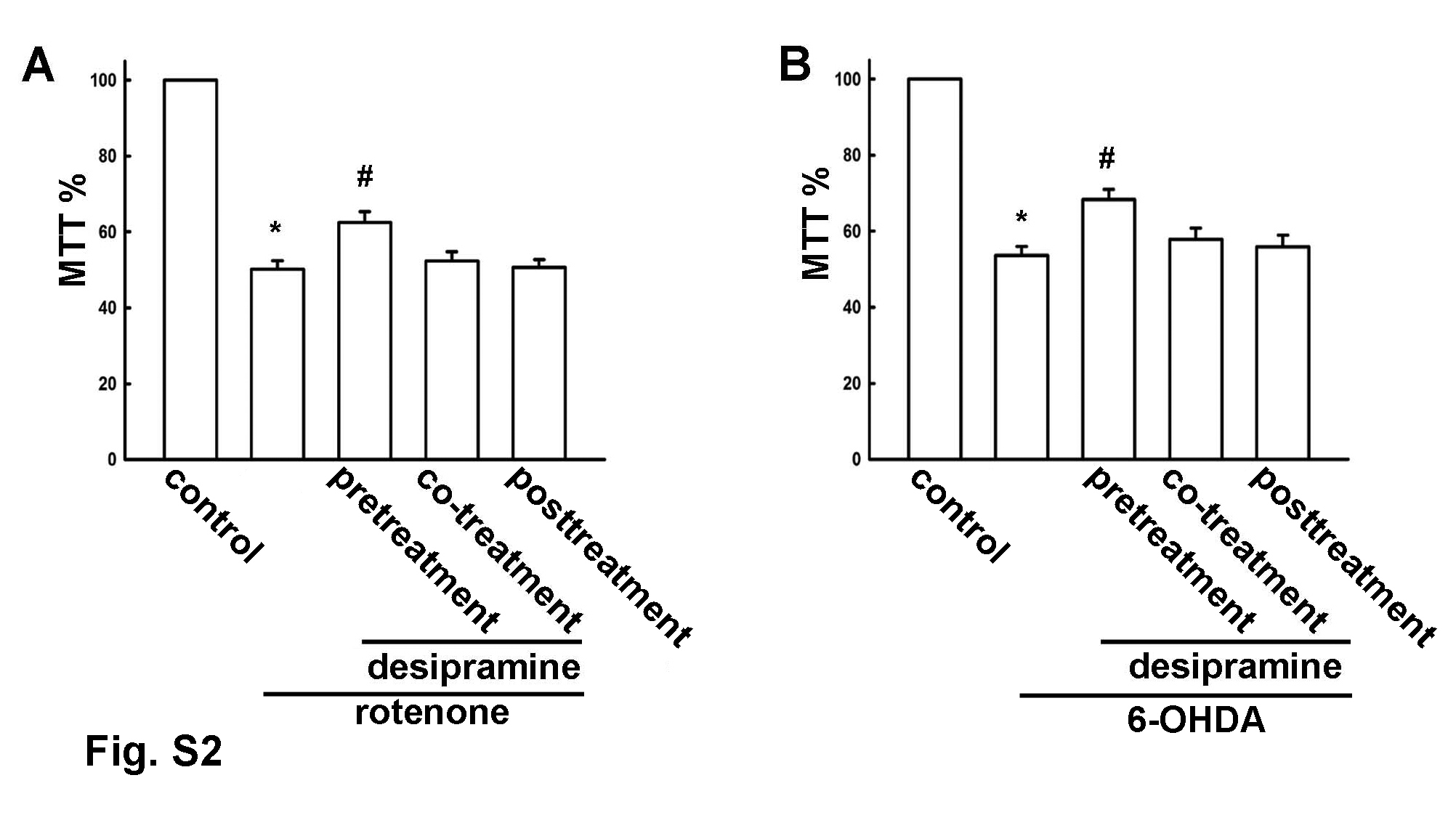

Supplement: Figure S2 — Protective effect of desipramine on rotenone- and 6-OHDA-induced neurotoxicity. Mes23.5 cells were treated with desipramine before (0 or 5 h) or after (5 h) rotenone (3 µM; A) or 6-OHDA (50 µM; B) for another 16 h. The cell viability was determined by MTT assay. Results are expressed as the means ± S.E.M. from four independent experiments. *, p<0.05 as compared with the vehicle control group. #, p<0.05 as compared with the rotenone- or 6-OHDA-treated group. (TIF) [file pone.0050138.s002.tif]
